# Neutrophil extracellular traps induce chemoresistance in human breast cancer cells through the PI3K/AKT/NF-κB pathway

**DOI:** 10.3389/fimmu.2026.1912140

**Published:** 2026-09-10

**Authors:** Juliana L. Souza, Vitor H. Almeida, Karina Martins-Cardoso, Luciana D. T. Carneiro, Vitória R. D. Azevedo, Gabriela Nestal de Moraes, Robson Q. Monteiro

**Affiliations:** 1 Laboratório de Trombose, Inflamação e Câncer, Instituto de Bioquímica Médica Leopoldo de Meis, Universidade Federal do Rio de Janeiro (UFRJ), Rio de Janeiro, Brazil; 2 Laboratório de Sinalização e Biologia Tumoral, Instituto de Bioquímica Médica Leopoldo de Meis, Universidade Federal do Rio de Janeiro (UFRJ), Rio de Janeiro, Brazil

**Keywords:** breast cancer, chemoresistance, doxorubicin, neutrophil extracellular traps (NETs), PI3K/AKT/NF-κB signaling, tumor microenvironment

## Abstract

**Introduction:**

Breast cancer is the most common malignancy and the leading cause of cancer-related mortality among women, and resistance to chemotherapy remains a major clinical challenge. Although doxorubicin is widely used, its efficacy is limited by chemoresistance. Neutrophil extracellular traps (NETs), web-like structures composed of DNA and proteins released by activated neutrophils, have been implicated in tumor progression and metastasis; however, their role in chemoresistance remains poorly understood. Here, we investigated the impact of NETs on doxorubicin resistance in human breast cancer cell lines.

**Methods:**

NETs were isolated from healthy donor blood and used to treat tumor cell lines (MCF7, T47D, and MDA-MB-231) prior to chemotherapy exposure. Cell viability and clonogenic potential were assessed by MTT and colony formation assays, while apoptotic signaling and AKT activation were evaluated by qPCR and Western blot. Pathway involvement was investigated using pharmacological inhibitors of PI3K, AKT, and NF-κB and siRNA-mediated AKT1 knockdown.

**Results:**

NETs were not cytotoxic but significantly increased clonogenic capacity and conferred resistance to doxorubicin. This effect was independent of the DNA scaffold and abolished by heat denaturation, implicating heat-labile, NET-associated components (most likely proteins) rather than the DNA backbone. NET exposure downregulated the pro-apoptotic factor BAX and upregulated the anti-apoptotic factor BCL2, indicating modulation of apoptosis-related factors toward a pro-survival profile. NETs induced AKT phosphorylation in the luminal cell lines and engaged the PI3K/AKT/NF-κB signaling axis, consistent with the modulation of downstream apoptotic factors. Inhibition of this pathway or AKT1 knockdown reversed the NET-induced chemoresistant phenotype, restoring chemosensitivity and apoptotic gene expression.

**Discussion:**

Collectively, these findings identify heat-labile, NET-associated components as mediators of breast cancer chemoresistance and support targeting NETs or their downstream signaling pathways to improve chemotherapy response.

## 1 Introduction

Breast cancer remains the most commonly diagnosed malignancy among women and a leading cause of cancer-related deaths worldwide, with incidence and mortality still rising despite significant therapeutic progress (1, 2). Although early detection and multimodal therapies have improved patient survival, recurrence and metastatic disease remain critical barriers to long-term outcomes and quality of life (3, 4). Conventional treatments — surgery, radiotherapy, hormone therapy, targeted therapy, and chemotherapy — form the therapeutic backbone. Nevertheless, intrinsic and acquired resistance to cytotoxic agents remains a central obstacle to durable responses, and although anthracyclines, taxanes, and platinum-based regimens are widely used, tumor heterogeneity and adaptive signaling frequently culminate in therapeutic failure (5–9).

Chemoresistance arises from multifactorial mechanisms, including drug efflux, enhanced DNA repair, suppressed apoptosis, metabolic alterations, and the emergence of cancer stem-like populations (10, 11). Beyond intrinsic cellular alterations, the tumor microenvironment (TME) has emerged as a critical determinant of drug response. The TME comprises a dynamic milieu of stromal, endothelial, and immune cells embedded within an extracellular matrix, whose interactions with cancer cells generate a protective niche that fosters chemoresistance by activating pro-survival signaling, promoting immune evasion, and favoring tumor dormancy (12–16).

Among TME immune components, neutrophils have emerged as versatile regulators of tumor progression. Tumor-associated neutrophils (TANs) display remarkable plasticity, ranging from anti-tumor (N1) to pro-tumor (N2) phenotypes depending on cytokine cues and tumor stage (17–19). Through angiogenesis, matrix remodeling, and immunosuppression, TANs actively facilitate tumor growth and metastatic dissemination (20, 21). Notably, high neutrophil infiltration in breast cancer correlates with poor prognosis and reduced therapeutic response (22–24).

A key neutrophil effector mechanism is the formation of neutrophil extracellular traps (NETs) — chromatin structures decorated with proteases and histones, first described by Brinkmann et al. (2004) (25). While NETs are essential for host defense, their aberrant formation within tumors has been increasingly associated with inflammation-driven progression, metastasis, and thrombosis (26, 27). NETs can entrap circulating tumor cells, promote epithelial–mesenchymal transition (EMT), and activate stromal components (28–30). Our group has previously demonstrated that NETs endow breast cancer cells with pro-metastatic properties by upregulating tissue factor, triggering PAR2 signaling, and inducing EMT-associated gene expression (29, 31). Recent evidence further indicates that NETs promote resistance to chemotherapy and radiotherapy by modulating the TGF-β axis and extracellular signaling (32–34); however, the precise molecular mechanisms by which NETs confer chemoresistance remain largely underexplored.

In this context, we evaluated the contribution of NETs to breast cancer chemoresistance. We show that NETs increase clonogenic capacity and confer doxorubicin resistance in human breast cancer cells in a DNA-independent, heat-sensitive manner. NET exposure modulated apoptosis-related factors toward a pro-survival profile, downregulating Bax and upregulating Bcl-2, effects mediated through activation of the PI3K/AKT/NF-κB signaling axis. Pharmacological inhibition and AKT1 knockdown both restored chemosensitivity, highlighting this pathway as a potential therapeutic target to overcome NET-driven chemoresistance.

## 2 Materials and methods

### 2.1 Cell lines and culture conditions

The experiments were conducted *in vitro* using the breast cancer cell lines MCF7 (RRID: CVCL_0031), T47D (RRID: CVCL_0553) (luminal A subtypes), and MDA-MB-231 (RRID: CVCL_0062) (triple-negative subtype) obtained from the Rio de Janeiro Cell Bank (Rio de Janeiro, RJ, Brazil). All cell lines tested negative for mycoplasma contamination by PCR prior to experimentation. Cells were cultured in Dulbecco’s Modified Eagle Medium (DMEM; Thermo Fisher Scientific, Waltham, MA, USA) supplemented with 10% fetal bovine serum (FBS; Gibco, Thermo Fisher Scientific) and 1% penicillin/streptomycin (Thermo Fisher Scientific). Cultures were maintained at 37 °C in a humidified atmosphere containing 5% CO_2_. Unless otherwise specified, cells were seeded at a density of 1 × 10^6^ cells per 25 cm² flask and allowed to adhere overnight before experimental procedures.

### 2.2 Isolation of neutrophils and NETs preparation

Peripheral blood was collected from healthy human donors after informed consent, in accordance with institutional ethical guidelines (approval number 82933518.0.0000.525, Hospital Universitário Clementino Fraga Filho, UFRJ), using sodium citrate (3.8%) as an anticoagulant, at a 1:9 v/v proportion. Neutrophils were purified through density gradient centrifugation using Histopaque-1077 (Merck, Darmstadt, Germany). To induce NETosis, neutrophils were stimulated with 500 nM phorbol 12-myristate 13-acetate (PMA; Sigma-Aldrich, St. Louis, MO, USA) for 3 h at 37 °C. The resulting NETs were harvested according to the simplified protocol described by Najmeh et al. (35). Briefly, the PMA-containing supernatant was completely removed, and the adherent NET layer was delicately washed twice with sterile room-temperature PBS to minimize carryover of residual PMA before collection. NETs were then recovered in PBS after gentle agitation and centrifuged at 400 × g for 10 min at 4 °C to remove cellular debris; the supernatant was collected and centrifuged again at 18,000 × g for 10 min at 4 °C, and the resulting NET pellet was resuspended in sterile PBS. DNA concentration of isolated NETs was measured using 1 μL of sample on a NanoDrop Lite spectrophotometer (Thermo Fisher Scientific, Waltham, MA, USA) at 260 nm, and preparations were stored at 4 °C for no longer than 24 h. NET preparations were operationally standardized by their extracellular DNA content; protein content was not independently quantified, and the stated NET concentrations therefore refer to NET-associated DNA rather than to total NET-associated protein. NET preparations obtained with this protocol were previously characterized by immunofluorescence, showing colocalization of extracellular DNA (DAPI) with citrullinated histone H3 (31). When indicated, NETs were boiled (95 °C, 10 min) to denature heat-labile components or treated with recombinant human DNase I (Pulmozyme^®^, Roche, Basel, Switzerland; 50 U/mL, 30 min) to degrade chromatin scaffolds.

### 2.3 Pharmacological inhibitors

To evaluate the contribution of PI3K/AKT/NF-κB signaling, cells were pretreated for 1 h prior to NETs exposure with the following inhibitors: LY294002 (PI3K inhibitor, 25 µM; Merck), MK-2206 (AKT inhibitor, 10 µM; Selleckchem, Houston, TX, USA), and BAY 11-7085 (NF-κB inhibitor, 10 µM; Sigma-Aldrich). Stock solutions were diluted in culture medium immediately before use.

### 2.4 Small interfering RNA-mediated AKT knockdown

To assess the involvement of AKT signaling, MDA-MB-231 cells were transfected with siRNA targeting AKT1 (AM16704, Ambion^®^, Thermo Fisher Scientific, Waltham, MA, USA) or a non-silencing control (NSC) siRNA (4390847, Ambion^®^, Thermo Fisher Scientific) for 48 hours using Lipofectamine RNAiMAX (Thermo Fisher Scientific) following the manufacturer’s protocol. During the transfection, at the 24-hour mark, NETs were added. After the transfection was completed, cells were counted and separated for the experiments which followed.

### 2.5 Cell viability assay (MTT)

For MTT assays, cells were seeded at 1 × 10^4^ per well in 96-well plates and incubated overnight. Initially, cells were exposed to NETs at concentrations of 250, 500, and 1000 ng/mL for 24 or 48 h in serum-free medium to determine the working concentration; all subsequent experiments used 500 ng/mL for 24 h. Where indicated, pharmacological inhibitors were added 1 h prior to NET exposure. Alternatively, cells were subsequently treated with doxorubicin for 48 h (Sigma-Aldrich) at IC_50_ (Half Maximal Inhibitory Concentration) concentrations. 3 h before the end of treatment, cells were incubated with 0.5 mg/mL MTT reagent (Sigma-Aldrich) at 37 °C. Formazan crystals were solubilized in DMSO, and absorbance was measured at 538 nm using a SpectraMax ABS Plus microplate reader (Molecular Devices, San Jose, CA, USA). Results were expressed as mean ± standard deviation of three independent experiments, considering normalization of untreated controls to 100%.

### 2.6 Colony formation assay

To assess long-term clonogenicity, 2,000 cells were plated per well in 6-well plates and incubated overnight. Cells were then exposed to 500 ng/mL NETs in medium supplemented with 1% FBS for 24 h, and subsequently treated with doxorubicin in complete medium for additional 48 h. In selected experiments, cells were pretreated with the pharmacological inhibitors 1 h prior to NET exposure. Cultures were maintained in complete medium for 13 days, replaced every 3–4 days. Colonies were stained with 0.1% crystal violet (Sigma-Aldrich) for 30 min, washed with PBS, photographed, and solubilized in 1 mL methanol. Absorbance was measured at 595 nm using a SpectraMax ABS Plus microplate reader (Molecular Devices, San Jose, CA, USA). Results were expressed as mean ± standard deviation of three independent experiments, with all conditions normalized to the single untreated, NET-free control (defined as 100%).

### 2.7 Quantitative real-time PCR

Cells were seeded at a density of 1 × 10^6^ cells per 25 cm² flask and incubated overnight. Then, they were starved in serum-free medium for 16 h, treated with NETs (500 ng/mL, 24 h), with or without inhibitor pretreatment. Total RNA was extracted using TRIzol reagent (Thermo Fisher Scientific) and quantified using 1 μL of sample on a NanoDrop Lite spectrophotometer (Thermo Fisher Scientific, Waltham, MA, USA). One microgram of RNA was treated with DNase I (Thermo Fisher Scientific) and reverse transcribed using the High-Capacity cDNA Reverse Transcription Kit (Applied Biosystems, Foster City, CA, USA). qPCR was performed using SYBR Green Master Mix (Thermo Fisher Scientific) on a StepOnePlus Real-Time PCR System. GAPDH was used as an endogenous control, and relative expression levels of BAX and BCL2 were calculated using the 2^-^ΔΔCT method. All primers used were acquired from Invitrogen, Carlsbad, CA, USA, and the sequences are provided in **Supplementary Table 1**.

### 2.8 Western blotting

Cells were seeded at a density of 1 × 10^6^ cells per 25 cm² flask and incubated overnight. Then, they were starved in serum-free medium for 16 h. For experiments measuring AKT phosphorylation, cells were treated with NETs (500 ng/mL) in serum-free medium for 15, 30, 60 min, 3 h, 6 h, and 24 h. For experiments measuring Bax and Bcl-2 protein levels, cells were stimulated with NETs (500 ng/mL) in serum-free medium for 24 h. Protein extracts were prepared in RIPA (Radioimmunoprecipitation) lysis buffer supplemented with protease and phosphatase inhibitors (Thermo Fisher Scientific) and quantified by the adapted Lowry method (Bio-Rad, Hercules, CA, USA). Equal amounts of protein (15–40 µg) were separated by SDS-PAGE (Sodium Dodecyl Sulfate-Polyacrylamide Gel Electrophoresis) (8–10%), transferred onto PVDF membranes (GE Healthcare, Chicago, IL, USA), and blocked with 5% non-fat milk. Membranes were incubated overnight at 4 °C with primary antibodies against total AKT, phospho-AKT, Bax, Bcl-2, and β-actin. After incubation with HRP-conjugated secondary anti-mouse or anti-rabbit antibodies, membranes were washed and the signal was developed. Information on antibodies used can be found on **Supplementary Table 2**. Immunoblots were detected using the ECL (Enhanced Chemiluminescence) reagent (GE Healthcare, Brazil) or Clarity Max (Bio-Rad, Brazil) and quantified by densitometry using ImageJ software.

### 2.9 Statistical analysis

Unless otherwise indicated in the corresponding figure legend, experiments were performed in at least three biological replicates. Statistical analyses were conducted using GraphPad Prism version 10.0 (GraphPad Software, San Diego, CA, USA), and the results were expressed as mean ± standard deviation. Comparisons between two conditions were assessed using the unpaired Student’s t-test. For analyses involving multiple conditions, one-way ANOVA followed by Tukey’s multiple comparisons post-test was applied. When two independent factors simultaneously influenced the dependent variable, two-way ANOVA followed by Šídák’s multiple comparisons post-test was used. Differences were considered statistically significant when P ≤ 0.05. The specific statistical test applied to each experiment is indicated in the corresponding figure legend. ns, not significant; *P < 0.05; **P < 0.01; ***P < 0.001; ****P < 0.0001.

## 3 Results

### 3.1 NETs do not reduce short-term viability but increase long-term colony growth of breast cancer cells

**Figure 1:**
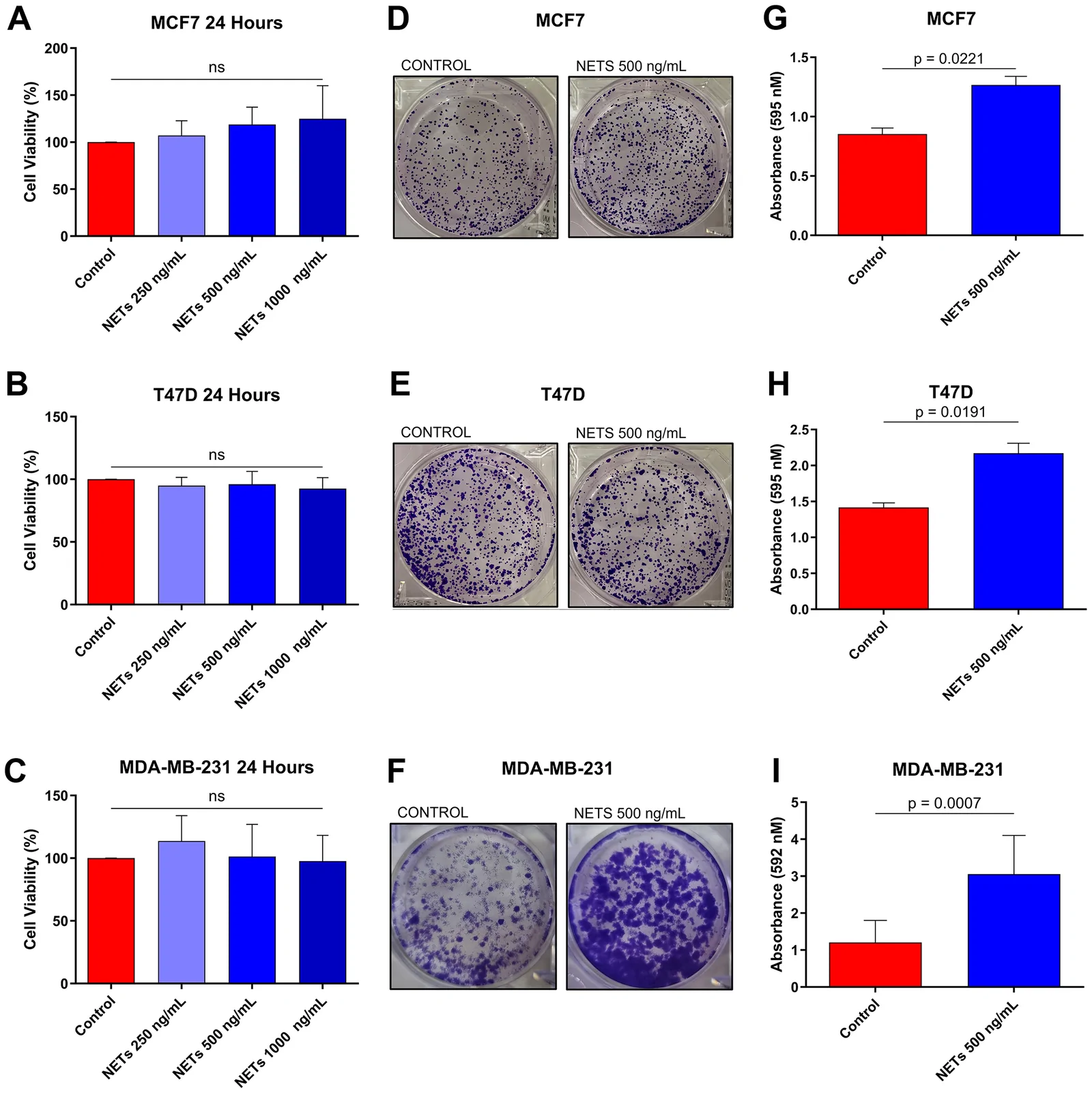
NETs alone do not hinder cell viability but enhance colony formation in breast cancer cells. **(A–C)** To assess the effects of NETs on cell viability and colony formation, MTT assays and Colony Formation Assays were performed. For the MTT assay, cells were seeded at 10,000 cells/well in 96-well cell culture plates. Then, cells were exposed to different concentrations of NETs (250, 500, and 1000 ng/mL) for 24 h in serum-free medium. After treatment, cells were incubated with a solution of MTT. The formazan crystals generated were solubilized in DMSO, and the optical density was measured at a wavelength of 538 nm. Cell viability was expressed as a percentage of the untreated, NET-free control. Values represent mean ± SD of four independent experiments. **(D–I)** For the clonogenic assay, cells were seeded at a low density of 2,000 cells/well in 6-well cell culture plates. Then, they were treated with NETs (500 ng/mL) for 24 h in medium supplemented with 1% fetal bovine serum (FBS). Subsequently, cultures were maintained in complete medium and allowed to grow for 13 days. Then, colonies were stained with crystal violet, photographed, crystal violet was eluted in methanol, and absorbance at 595 nm was measured as a readout of colony-associated biomass. **(D–F)** Representative image of three independent experiments of colonies formed by each cell line. **(G–I)** Crystal violet quantification. *C*onditions were normalized to the single untreated, NET-free control (defined as 100%). Values represent mean ± SD of four independent experiments. For MTT assay analysis, one-way ANOVA and Tukey’s post-test were used; for colony formation assay analysis, Student’s t-test was performed. ns, not significant.

NETs have been shown to contribute to tissue damage in various inflammatory conditions, including cancer and autoimmune diseases, by inducing cytotoxic effects in healthy tissues (36, 37). To investigate whether NETs could elicit a similar effect on our models, we treated MCF7, T47D, and MDA-MB-231 cell lines with NETs in increasing concentrations (250, 500, and 1000 ng/mL) for 24 or 48 h. Cell viability was assessed using the MTT assay, which revealed no significant decrease in viability across the tested conditions (**Figures 1A–C**; **Supplementary Figure 1**), indicating that NETs do not intrinsically damage these cells. Of note, extended exposure for 48 h significantly increased MCF7 cell viability at 1000 ng/mL, pointing to a potential pro-survival effect at higher concentrations and longer time points (**Supplementary Figure 1**).

Furthermore, we performed colony formation assays to evaluate the impact of NETs on the clonogenic potential of breast cancer cells. NET-treated cells showed a significant increase in colony formation across all three cell lines (**Figures 1D–F**). Quantitative analysis of the crystal violet staining confirmed the enhanced clonogenic capacity induced by NETs (**Figures 1G–I**), suggesting that NETs provide a proliferative advantage to breast cancer cells.

### 3.2 NETs promote resistance to doxorubicin

**Figure 2:**
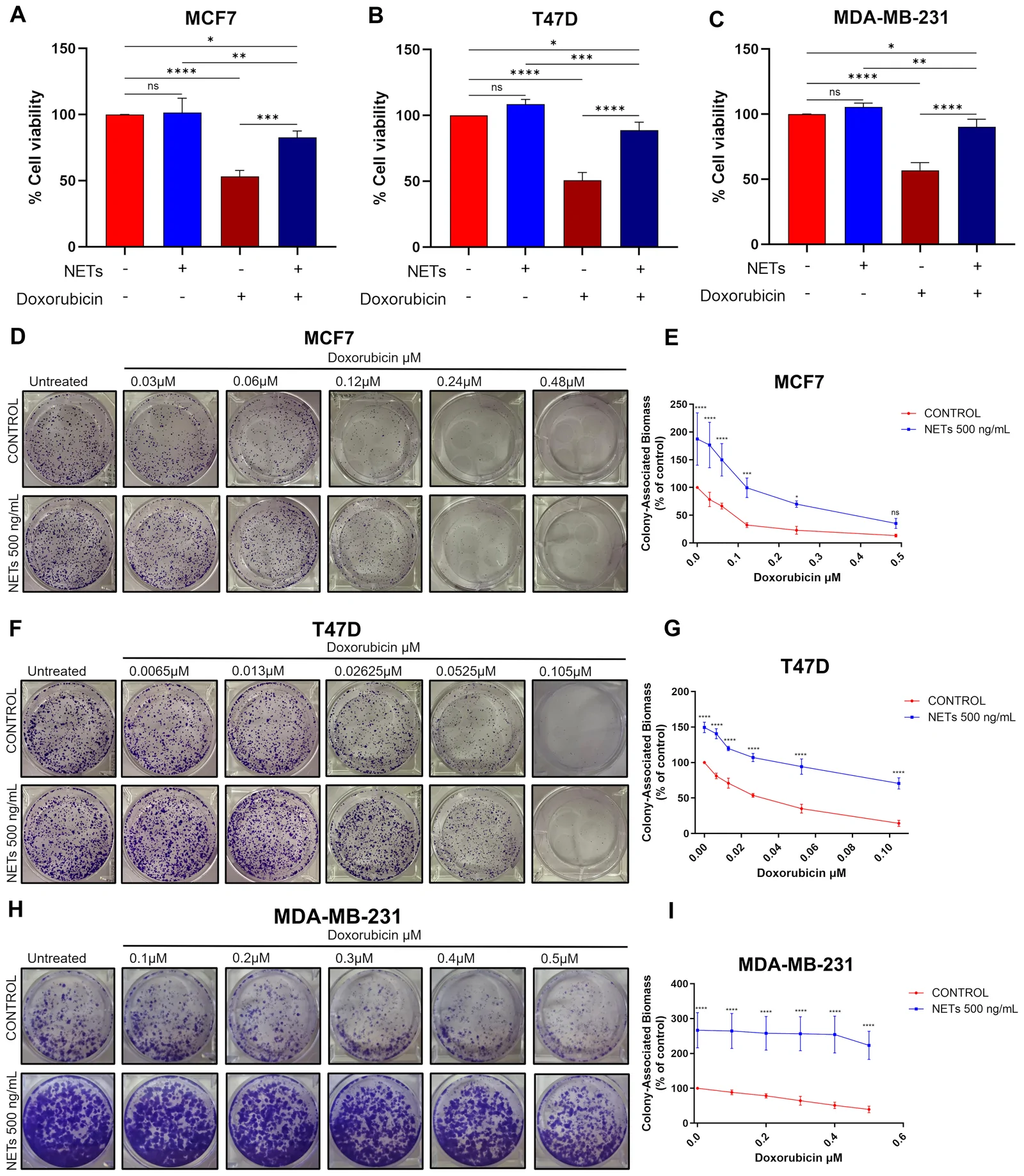
NETs reduce the sensitivity of breast cancer cells to doxorubicin. To assess the effects of NETs on cell resistance to doxorubicin, MTT assays and Colony Formation Assays were performed. **(A–C)** For the MTT assay, cells were seeded at 10,000 cells/well in 96-well cell culture plates. Then, cells were exposed to NETs (500 ng/mL) for 24 h in serum-free medium. After this stimulus, cells were treated with doxorubicin at IC_50_ concentrations, previously determined for each cell line, for 48 h in complete medium. After treatment, cells were incubated with a solution of MTT. The formazan crystals generated were solubilized in DMSO, and the optical density was measured at a wavelength of 538 nm. Cell viability was expressed as a percentage of the untreated, NET-free control. Values represent mean ± SD of three independent experiments. **(D–I)** For the colony formation assay, cells were seeded at a low density of 2,000 cells/well in 6-well cell culture plates. Then, they were stimulated with NETs (500 ng/mL) for 24 h in medium supplemented with 1% fetal bovine serum (FBS). Subsequently, cells were treated with doxorubicin at different concentrations for 48 h in complete medium, then allowed to grow for 13 days. Then, colonies were stained with crystal violet, photographed, crystal violet was eluted in methanol, and absorbance at 595 nm was measured as a readout of colony-associated biomass. **(D, F, H)** Representative image of three independent experiments of colonies formed by each cell line. **(E, G, I)** Crystal violet quantification. Conditions were normalized to the single untreated, NET-free control (defined as 100%). Values represent mean ± SD of three independent experiments. For MTT assay analysis, one-way ANOVA and Tukey’s post-test were used; for colony formation assay analysis, two-way ANOVA and Šídák’s multiple comparisons post-test were used. *P < 0.05; **P < 0.01; ***P < 0.001; ****P < 0.0001.

We next examined whether NET exposure could influence the response to chemotherapy. Cells were pretreated with NETs (500 ng/mL, 24 h), subsequently exposed to doxorubicin for 48 h, and assessed for viability. Across all three breast cancer cell lines, MTT assays demonstrated a marked resistance to the chemotherapeutic agent in NET-pretreated as compared to untreated cells (**Figures 2A–C**). Confirming these data, colony-formation assays showed robust clonogenic capacity across increasing doxorubicin concentrations in all three cell lines. This was evident both in the representative crystal-violet images (**Figures 2D, F, H**) and in their corresponding quantifications (**Figures 2E, G, I**), where NET-treated cells consistently retained a higher colony-associated biomass than untreated controls at matched doxorubicin doses. Together, these results establish that NETs confer a protective phenotype against doxorubicin, promoting chemoresistance in distinct molecular subtypes of breast cancer.

### 3.3 NET-associated chemoprotection is DNA-independent and heat-sensitive

**Figure 3:**
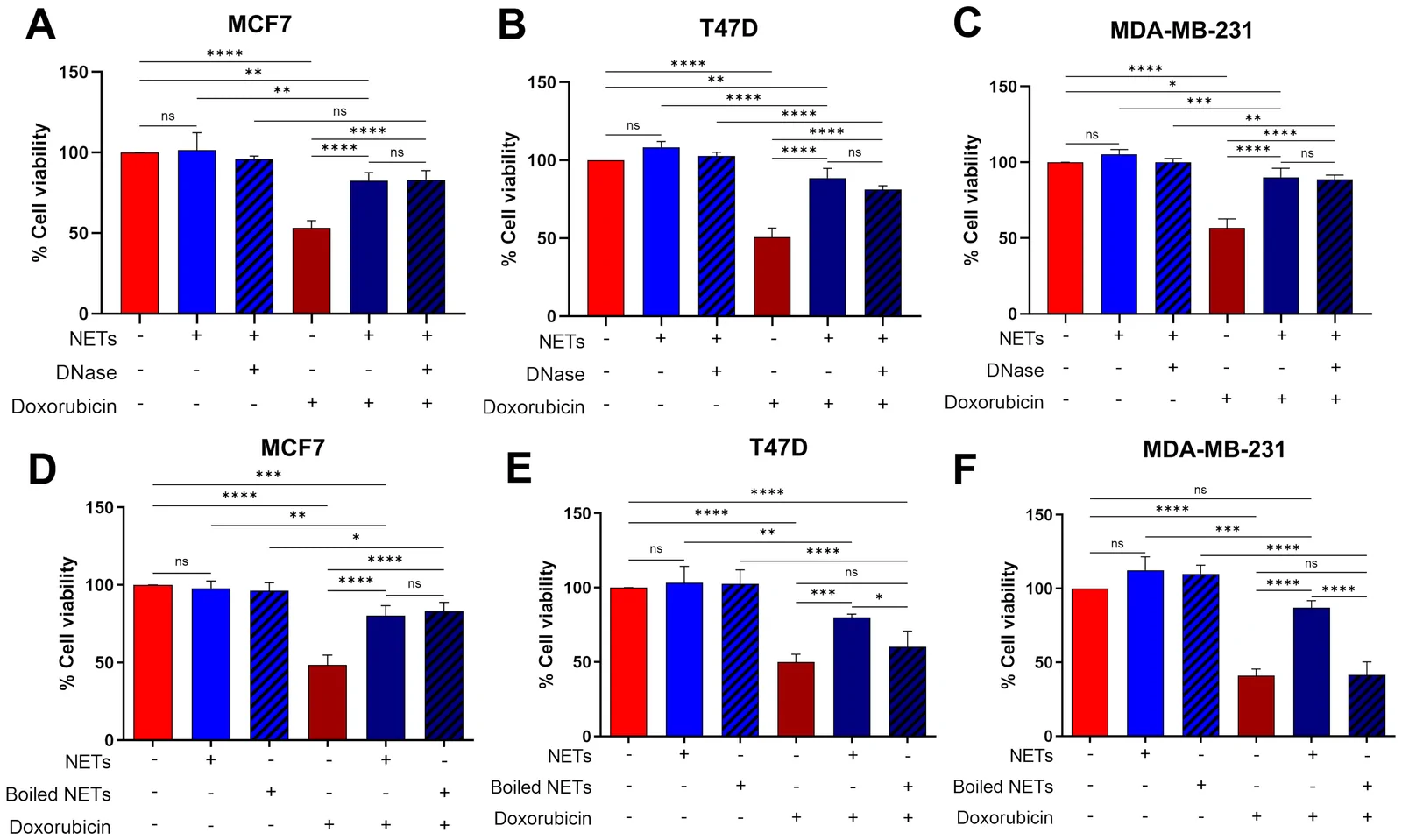
NET-associated chemoprotection is DNA-independent and heat-sensitive. To assess which NETs components are responsible for the acquisition of resistance to doxorubicin, NETs were treated with recombinant human DNase I (50 U/mL) for 30 min or boiled for 10 min at 95 °C prior to use, and MTT assays were performed. Cells were seeded at 10,000 cells/well in 96-well cell culture plates. Then, cells were exposed to NETs (500 ng/mL), DNase-treated NETs (500 ng/mL) **(A–C)**, or boiled NETs (500 ng/mL) **(D–F)** for 24 h in serum-free medium. After stimuli, cells were treated with doxorubicin at IC_50_ concentrations, previously determined for each cell line, for 48 h in complete medium. After treatment, cells were incubated with a solution of MTT. The formazan crystals generated were solubilized in DMSO, and the optical density was measured at a wavelength of 538 nm. Cell viability was expressed as a percentage of the untreated, NET-free control. Values represent mean ± SD of three independent experiments. One-way ANOVA and Tukey’s post-test were used. ns, not significant; *P < 0.05; **P < 0.01; ***P < 0.001; ****P < 0.0001.

To dissect the molecular mediators responsible for NET-induced resistance, we compared the effects of intact NETs with those of NETs treated with DNase I. Interestingly, DNase-mediated digestion of the DNA scaffold did not abolish the chemoprotective effects (**Figures 3A–C**). Due to these findings, we performed the same experiment, now boiling NETs in order to denature heat-labile components. Heat-denaturation, on the other hand, fully suppressed NETs-mediated chemoresistance (**Figures 3D–F**), indicating that an intact DNA scaffold is not required and that the chemoprotective activity depends on one or more heat-labile, NET-associated components. Because boiling is a nonspecific treatment, these experiments do not establish the molecular identity of the effector; nonetheless, together they implicate heat-labile NET-associated components, rather than the chromatin scaffold, as the critical effectors underlying the chemoresistant phenotype in breast cancer cell lines.

### 3.4 NETs modulate the expression of apoptosis-related factors

**Figure 4:**
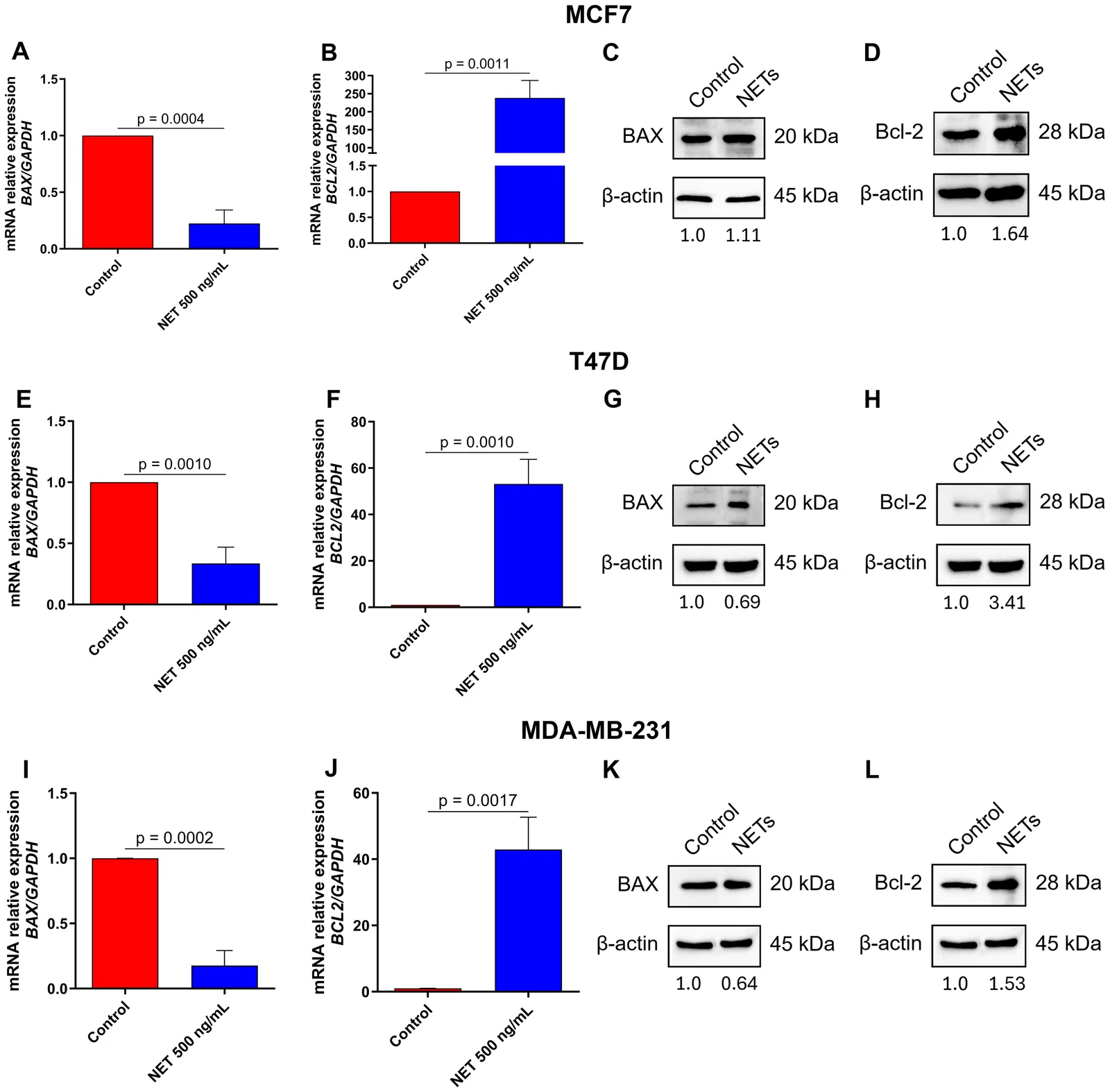
NETs modulate the expression of apoptotic pathway components, promoting a pro-survival phenotype. To assess the mechanisms behind the acquisition of resistance to doxorubicin, qPCR and Western blotting for apoptotic proteins were performed, with cells starved for 16 h, treated with NETs (500 ng/mL) for 24 h, and then samples were collected for each assay. For the qPCR, after treatment, mRNA was extracted and converted into cDNA. Gene expression assays for BAX **(A, E, I)** and BCL2 **(B, F, J)** were performed. The comparative CT method (ΔΔCT) was utilized to calculate relative mRNA expression, and GAPDH was used as the reference gene. Values represent mean ± SD of three independent experiments. Student’s t-test was performed, and P values are as indicated in each graph. For the Western blotting, cells were lysed, and levels of Bax **(C, G, K)**, Bcl-2 **(D, H, L)**, and β-actin (loading control) were determined. Representative image of two experiments, with densitometry levels indicated below.

Next, we examined the impact of NETs on the expression of apoptosis-related factors in breast cancer cells. Gene expression assessment by qPCR for upstream regulators of apoptosis demonstrated a substantial decrease in the expression of the proapoptotic factor *BAX* (**Figures 4A, E, I**) and an increase in the levels of the antiapoptotic factor *BCL2* (**Figures 4B, F, J**) across all cell lines. At the protein level, evaluated in two independent experiments, Western blot analysis showed an apparent increase in Bcl-2 in all three cell lines (**Figures 4D, H, L**). The Bax immunoblots suggested a trend toward reduction in T47D and MDA-MB-231 cells (**Figures 4G, K**), with no appreciable change observed in MCF7 cells (**Figure 4C**). Collectively, these findings suggest that NETs modulate apoptosis-related factors toward a profile associated with cell survival, though the extent of this effect varies across cell lines.

### 3.5 NET exposure induces time-dependent AKT phosphorylation and engages the PI3K/AKT/NF-κB pathway

**Figure 5:**
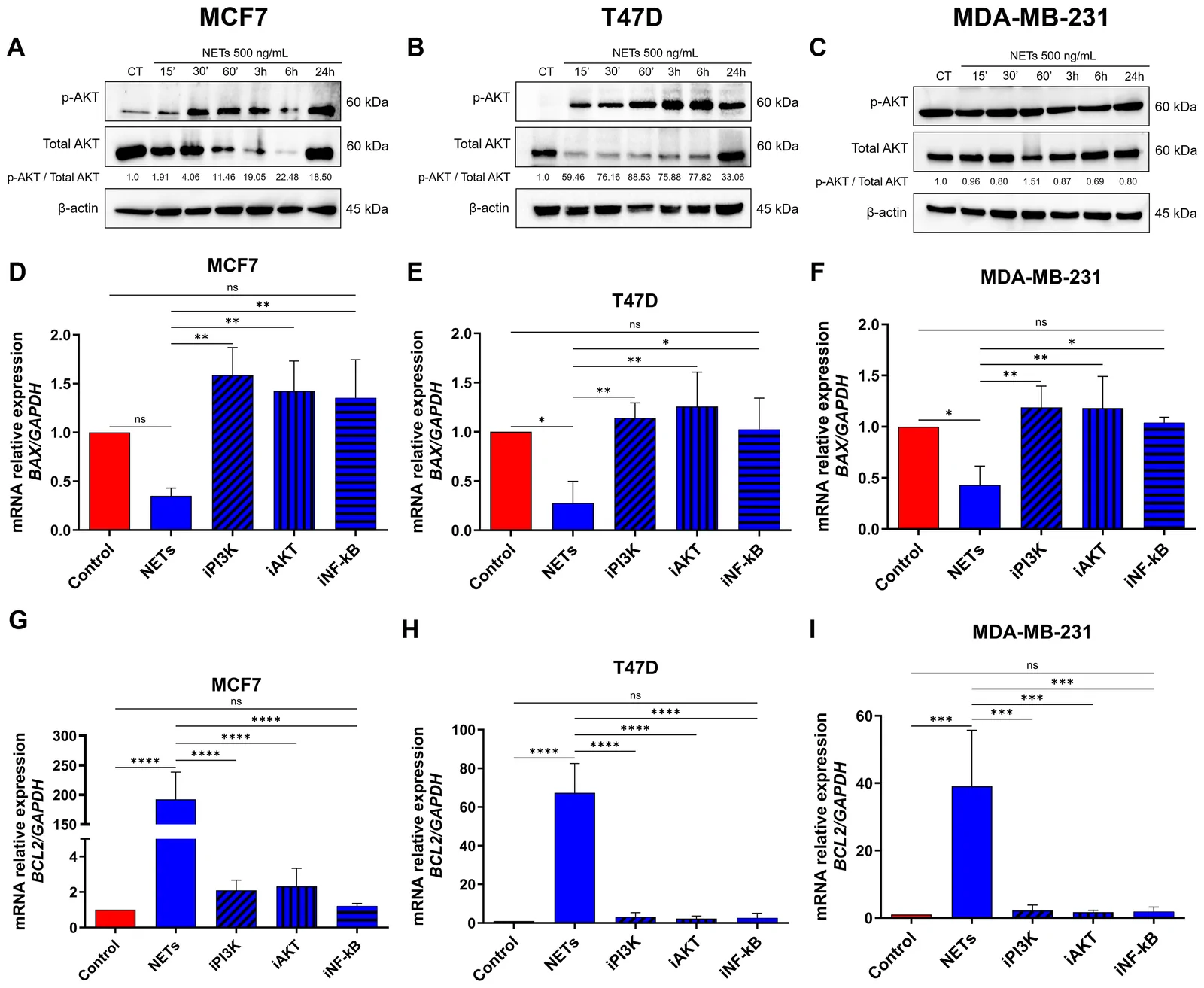
NETs activate AKT in luminal breast cancer cells and modulate apoptotic pathway components via PI3K/AKT/NF-κB signaling. To assess the mechanisms responsible for the modulation of the apoptotic components, Western blotting and qPCR were performed. **(A–C)** Cells were starved for 16 h, stimulated with NETs (500 ng/mL) for 15, 30, 60 minutes, 3, 6, or 24 h. Then, cells were lysed, and levels of total AKT, p-AKT, and β-actin (loading control) were determined by Western blotting. Representative image of two experiments, with densitometry levels indicated below. **(D–I)** For qPCR, cells were starved for 16 h, then treated with PI3K (LY294002 – 25 µM), AKT (MK-2206 – 10 µM), or NF-κB (BAY 11-7085 – 10 µM) inhibitors 1 h prior to NETs (500 ng/mL) exposure for 24 h. After stimuli, mRNA was extracted and converted into cDNA. Gene expression assays for BAX **(D–F)** and BCL2 **(G–I)** were performed. The comparative CT method (ΔΔCT) was utilized to calculate relative mRNA expression, and GAPDH was used as the reference gene. Values represent mean ± SD of three independent experiments. One-way ANOVA and Tukey’s post-test were used. ns, not significant; *P < 0.05; **P < 0.01; ***P < 0.001; ****P < 0.0001.

To further elucidate the mechanisms behind NET-induced chemoresistance, we investigated the role of the PI3K/AKT/NF-κB signaling pathway. This pathway is well-documented for its involvement in cellular survival, proliferation, and apoptosis regulation and is frequently deregulated in cancer, causing therapy resistance (38, 39). Time-course Western blots, quantified as the p-AKT/total-AKT ratio to account for changes in total AKT, revealed a rapid, time-dependent phosphorylation of AKT following treatment with NETs (500 ng/mL) in the MCF7 and T47D cell lines (**Figures 5A, B**), arising within 15–30 min, peaking between 30–60 min and remaining elevated for up to 24 h in T47D (**Figure 5B**). In contrast, MDA-MB-231 cells showed no appreciable change in the p-AKT/total-AKT ratio across the same time course (**Figure 5C**). In the luminal lines, these activation patterns are consistent with engagement of upstream receptor signaling by extracellular NET components, and the activation of PI3K. To assess the functional impact of AKT activation on apoptotic pathways, we utilized inhibitors targeting PI3K (LY294002), AKT (MK-2206), and NF-κB (BAY 11-7085) prior to NET exposure. qPCR analysis of pro-apoptotic BAX (**Figures 5D–F**) and anti-apoptotic BCL2 (**Figures 5G–I**) showed that these inhibitors significantly prevented NET-induced modulation of these genes across all breast cancer cell lines. Notably, this transcriptional dependence was observed in all three cell lines, including MDA-MB-231, indicating that NET-driven engagement of the PI3K/AKT/NF-κB axis in the triple-negative model operates even in the absence of a detectable increase in AKT phosphorylation by immunoblot. The reversal of gene expression patterns suggests that NETs modulate apoptotic pathways through the PI3K/AKT/NF-κB axis.

### 3.6 Pharmacological inhibition of PI3K/AKT/NF-κB reduces NETs-induced chemoresistance

**Figure 6:**
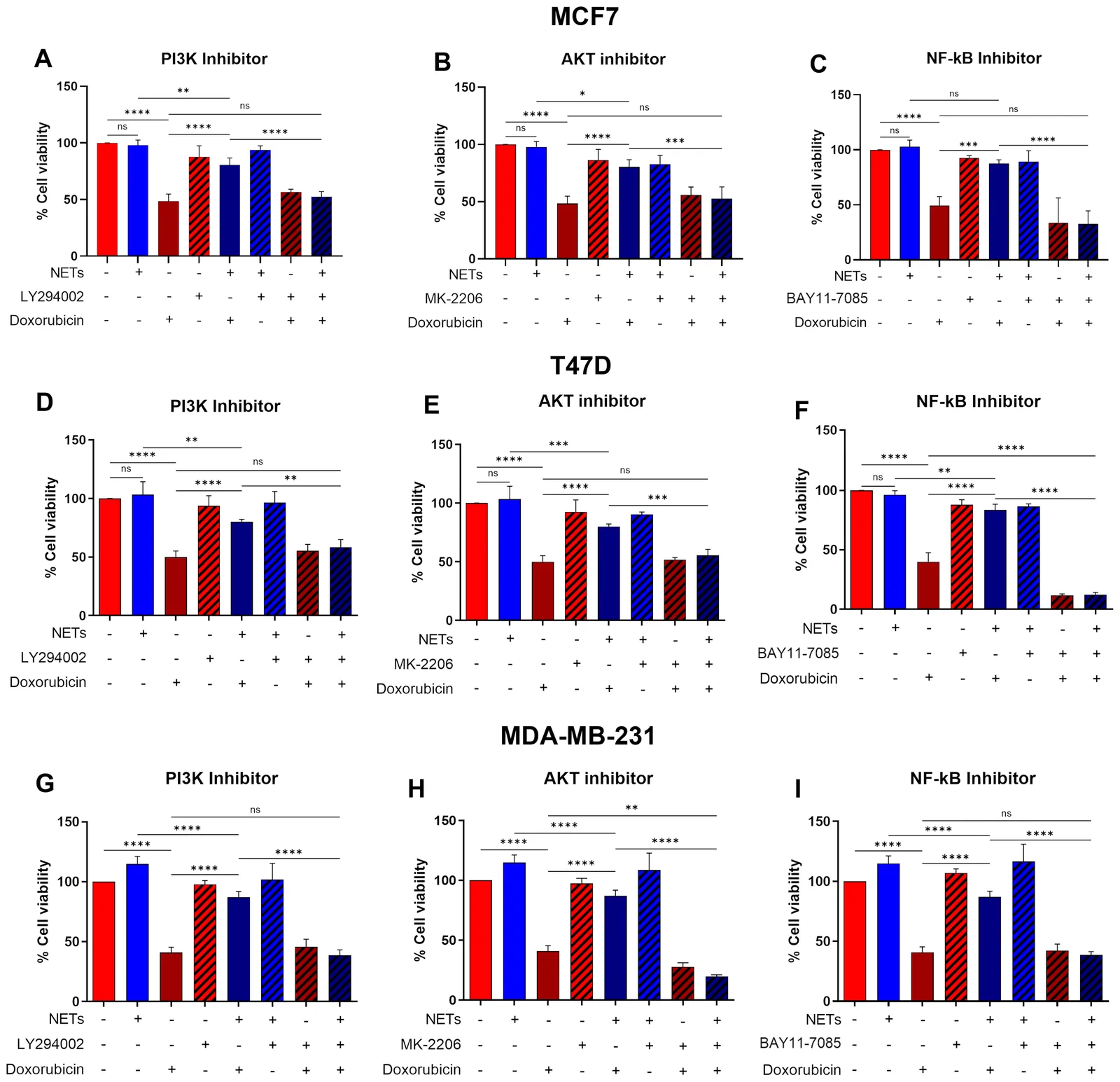
Inhibition of PI3K, AKT, or NF-κB reverses NET-induced resistance to doxorubicin. To assess the effects of PI3K/AKT/NF-κB pathway inhibition on the acquisition of resistance after NETs exposure, MTT assays were performed. Cells were seeded at 10,000 cells/well in 96-well cell culture plates. Then, cells were treated with **(A, D, G)** PI3K (LY294002 – 25 µM), **(B, E, H)** AKT (MK-2206 – 10 µM), or **(C, F, I)** NF-κB (BAY 11-7085 – 10 µM) inhibitors 1 h prior to NETs (500 ng/mL) exposure for 24 h in serum-free medium. After stimuli, cells were treated with doxorubicin at IC_50_ concentrations, previously determined for each cell line, for 48 h in complete medium. After treatment, cells were incubated with a solution of MTT. The formazan crystals generated were solubilized in DMSO, and the optical density was measured at a wavelength of 538 nm. Cell viability was expressed as a percentage of the untreated, NET-free control. Values represent mean ± SD of three independent experiments. One-way ANOVA and Tukey’s post-test were used. ns, not significant; *P < 0.05; **P < 0.01; ***P < 0.001; ****P < 0.0001.

**Figure 7:**
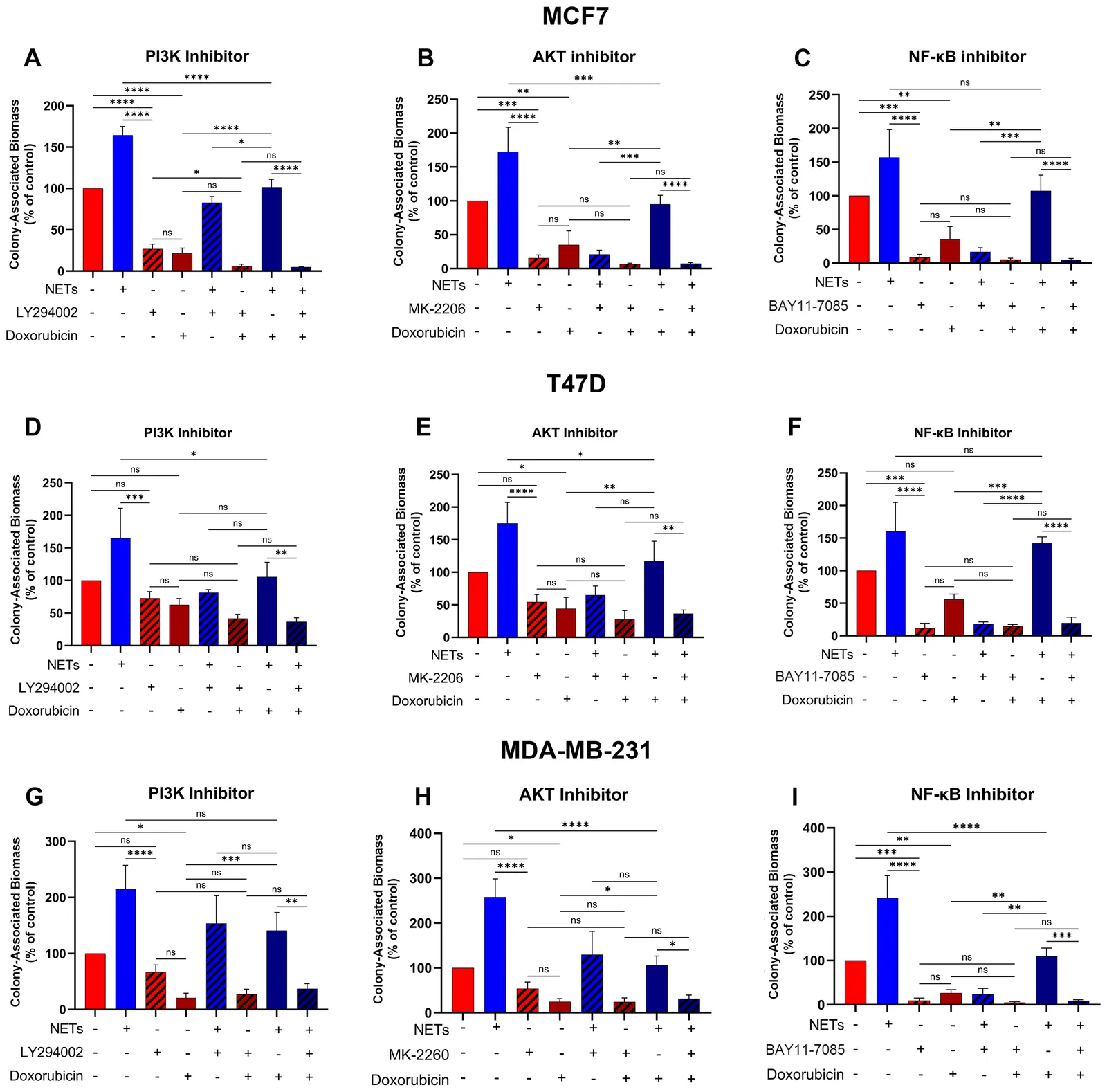
PI3K, AKT, and NF-κB inhibitors reverse NET-induced chemoresistance and colony formation in breast cancer cells. To assess the effects of PI3K/AKT/NF-κB pathway inhibition on the acquisition of resistance and clonogenic ability after NETs exposure, Colony Formation Assays were performed. Cells were seeded at a low density of 2,000 cells/well in 6-well cell culture plates. Then, cells were treated with **(A, D, G)** PI3K (LY294002 – 25 µM), **(B, E, H)** AKT (MK-2206 – 10 µM), or **(C, F, I)** NF-κB (BAY 11-7085 – 10 µM) inhibitors 1 h prior to NETs (500 ng/mL) exposure for 24 h in serum-free medium. After stimuli, cells were treated with doxorubicin for 48 h in complete medium, then allowed to grow for 13 days. Then, colonies were stained with crystal violet, photographed, crystal violet was eluted in methanol, and absorbance at 595 nm was measured as a readout of colony-associated biomass. Values represent mean ± SD of three independent experiments. One-way ANOVA and Tukey’s post-test were used. ns, not significant; *P < 0.05; **P < 0.01; ***P < 0.001; ****P < 0.0001.

To confirm the functional relevance of AKT signaling pathway on chemoresistance, we pretreated cells with inhibitors of PI3K (LY294002), AKT (MK-2206), or NF-κB (BAY 11-7085) prior to NETs exposure and doxorubicin treatment. MTT assay showed that pathway inhibition at these distinct levels reduced NETs-induced chemoresistance phenotype in MCF7 (**Figures 6A–C**), T47D (**Figures 6D–F**), and MDA-MB-231 cells (**Figures 6G–I**), with the magnitude of the effect varying across cell lines and inhibitors. To further substantiate these findings, we also performed colony formation assays in all cell lines. Consistently, inhibition of PI3K, AKT, or NF-κB significantly reduced colony-associated biomass in NET-treated cells, both with and without doxorubicin treatment (**Figure 7**; **Supplementary Figure 2**). These results confirm the critical role of PI3K/AKT/NF-κB signaling cascade in supporting the survival and proliferation of NETs-exposed cells, even in the presence of chemotherapy. The differential magnitude of these effects across MCF7, T47D, and MDA-MB-231 likely reflects subtype-specific basal pathway activity and compensatory signaling, consistent with a shared but not identical dependence on PI3K/AKT/NF-κB signaling.

### 3.7 AKT1 knockdown confirms its involvement in NET-associated chemoprotection in MDA-MB-231 cells

**Figure 8:**
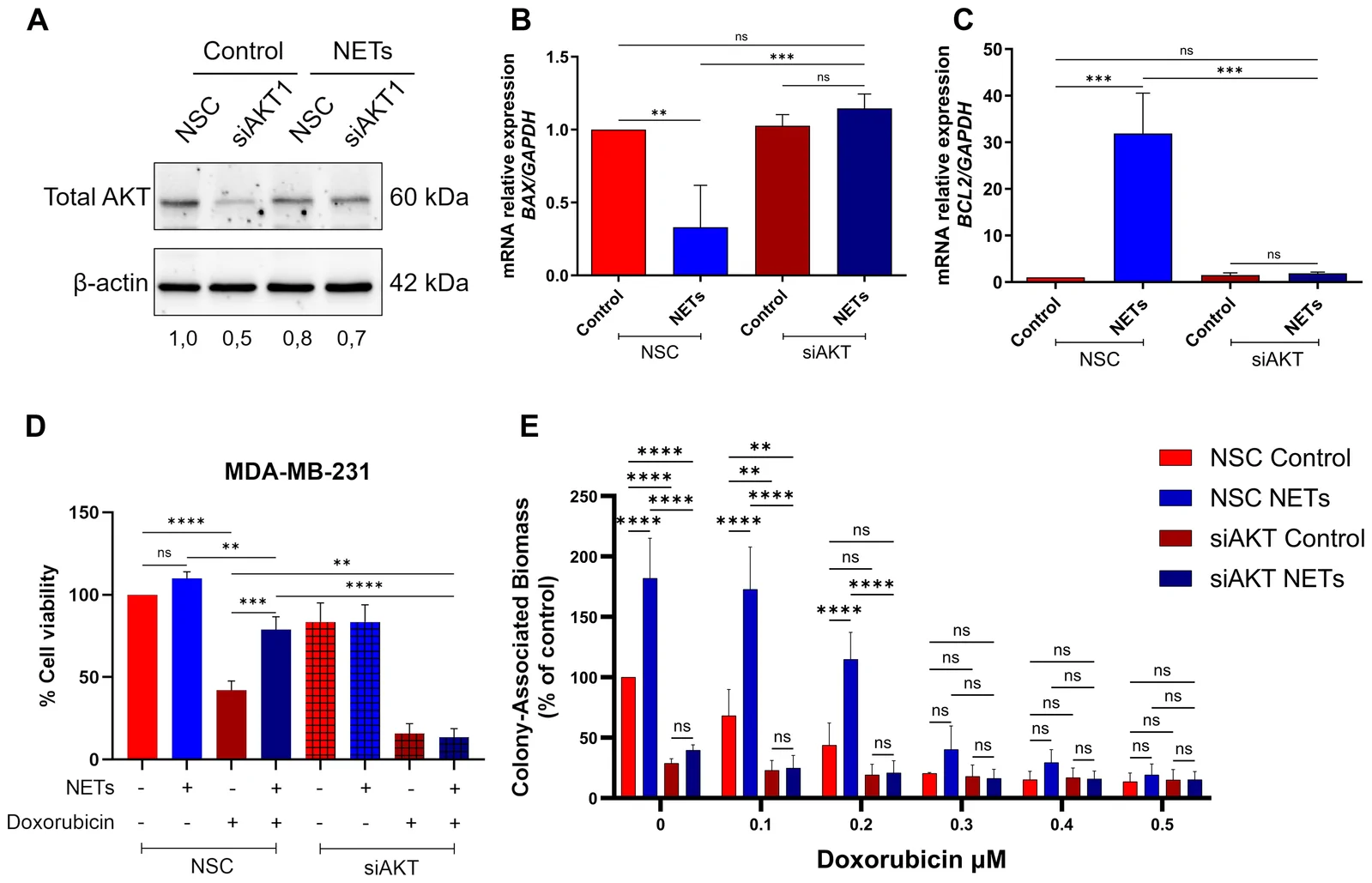
AKT1 knockdown impedes the protective effects of NETs. MDA-MB-231 cells were transfected with siAKT1 using Lipofectamine RNAiMAX for 48 h, with NETs (500 ng/mL) added at the 24-hour mark. After transfection, cells were counted and separated for each assay. NSC: non-silencing control; siAKT1: AKT1-targeting siRNA. **(A)** Levels of total AKT and β-actin (loading control) were determined by Western blotting. Representative image of three experiments, with densitometry values normalized to the NSC, NET-free control (set to 1.0) indicated below. **(B, C)** Gene expression assays for *BAX* **(B)** and *BCL2* **(C)** were performed by qPCR using the ΔΔCT method, with *GAPDH* as reference gene. Values represent mean ± SD of three independent experiments. **(D)** After transfection, cells were seeded at 10,000 cells/well and treated with doxorubicin at IC_50_ concentration for 48 h. Cell viability was assessed by MTT assay and expressed as a percentage of the control. Values represent mean ± SD of three independent experiments. **(E)** After transfection, cells were seeded at 2,000 cells/well, treated with increasing doxorubicin concentrations for 48 h, and allowed to grow for 13 days. Colonies were stained with crystal violet and quantified by absorbance at 595 nm as a readout of colony-associated biomass. Values represent mean ± SD of three independent experiments. *C*onditions were normalized to the single untreated, NET-free control (defined as 100%). For qPCR and MTT analyses, one-way ANOVA and Tukey’s post-test were used; for colony formation, two-way ANOVA and Šídák’s post-test were used. ns, not significant; **P < 0.01; ***P < 0.001; ****P < 0.0001.

To corroborate the pharmacological findings with a genetic approach, we performed siRNA-mediated knockdown of AKT1 — the central node of the pathway, downstream of PI3K and upstream of NF-κB — in the triple-negative cell line in MDA-MB-231, chosen as a clinically relevant proof-of-concept model. Western blot analysis confirmed the efficient AKT1 knockdown: relative to the NSC, NET-free control (set to 1.0), total AKT protein levels were reduced approximately 50% compared to NSC-transfected controls, and NET exposure did not restore total AKT to control levels (**Figure 8A**). With the technique validated, we next assessed whether AKT1 silencing would affect the cellular and molecular responses to NETs.

At the gene expression level, AKT1 knockdown prevented the NET-induced downregulation of *BAX* and upregulation of *BCL2* observed in NSC-transfected cells, with siAKT1 cells showing no significant modulation of either gene upon NET exposure (**Figures 8B, C**). These findings confirm that NETs modulate apoptotic gene expression through an AKT1-dependent mechanism.

Regarding cell number, NETs exposure did not significantly alter cell density in NSC-transfected cells compared to NSC controls. However, within the siAKT1 group, NET-treated cells showed a significantly higher cell count than untreated siAKT1 controls (**Supplementary Figures 3A, B**). In clonogenic assays, NETs significantly enhanced colony formation in NSC cells, while AKT1 knockdown markedly reduced this capacity. NET exposure in siAKT1 cells did not significantly rescue clonogenic potential compared to NSC controls (**Supplementary Figures 3C, D**). However, notably, within the siAKT1 group, NET-treated cells formed significantly more colonies than untreated siAKT1 controls (**Supplementary Figure 3E**), indicating that even in the context of AKT1 silencing, NETs retain a residual, albeit limited, pro-survival effect on clonogenic capacity.

In MTT assays, siAKT1-transfected cells treated with doxorubicin showed markedly reduced viability compared to NSC controls, and NET pre-exposure failed to confer the protective effect observed in NSC cells (**Figure 8D**). Consistently, clonogenic assays across a range of doxorubicin concentrations demonstrated that AKT1 knockdown reduced the NET-induced chemoresistant phenotype (**Figure 8E**; **Supplementary Figure 4**). Notably, the combination of AKT1 silencing and doxorubicin treatment produced a pronounced cytotoxic effect that effectively overrode the cytoprotective influence of NETs, further underscoring the involvement of AKT1 signaling in NET-driven chemoresistance.

Altogether, these findings identify AKT1 as a key mediator of NETs-induced survival signaling in breast cancer cells, with its loss substantially reducing both the enhanced clonogenicity and the chemoresistant phenotype conferred by NETs, consistent with the pharmacological inhibition of PI3K, AKT, and NF-κB, which likewise reduced NET-induced chemoresistance.

## 4 Discussion

In this study, we demonstrate that neutrophil extracellular traps (NETs) are potent mediators of chemoresistance in breast cancer, acting through activation of the PI3K/AKT/NF-κB signaling pathway and remodeling of apoptotic programs. Our data reveal that NETs do not exert direct cytotoxicity on tumor cells but rather promote a pro-survival phenotype characterized by increased clonogenic potential and reduced susceptibility to doxorubicin. The protective effect was abolished by heat treatment but not by DNase-mediated chromatin degradation, implicating heat-labile, NET-associated components – most likely proteins – rather than the DNA scaffold, as the functional effectors for the acquisition of resistance. These findings expand the growing body of evidence linking NETs to therapy evasion in several cancer contexts, as NET components such as neutrophil elastase, myeloperoxidase, cathepsin G, and matrix metalloproteinases (MMP-9, MMP-2) have been shown to modify tumor cell signaling, extracellular matrix composition, and drug penetration (36, 37, 40–42).

**Figure 9:**
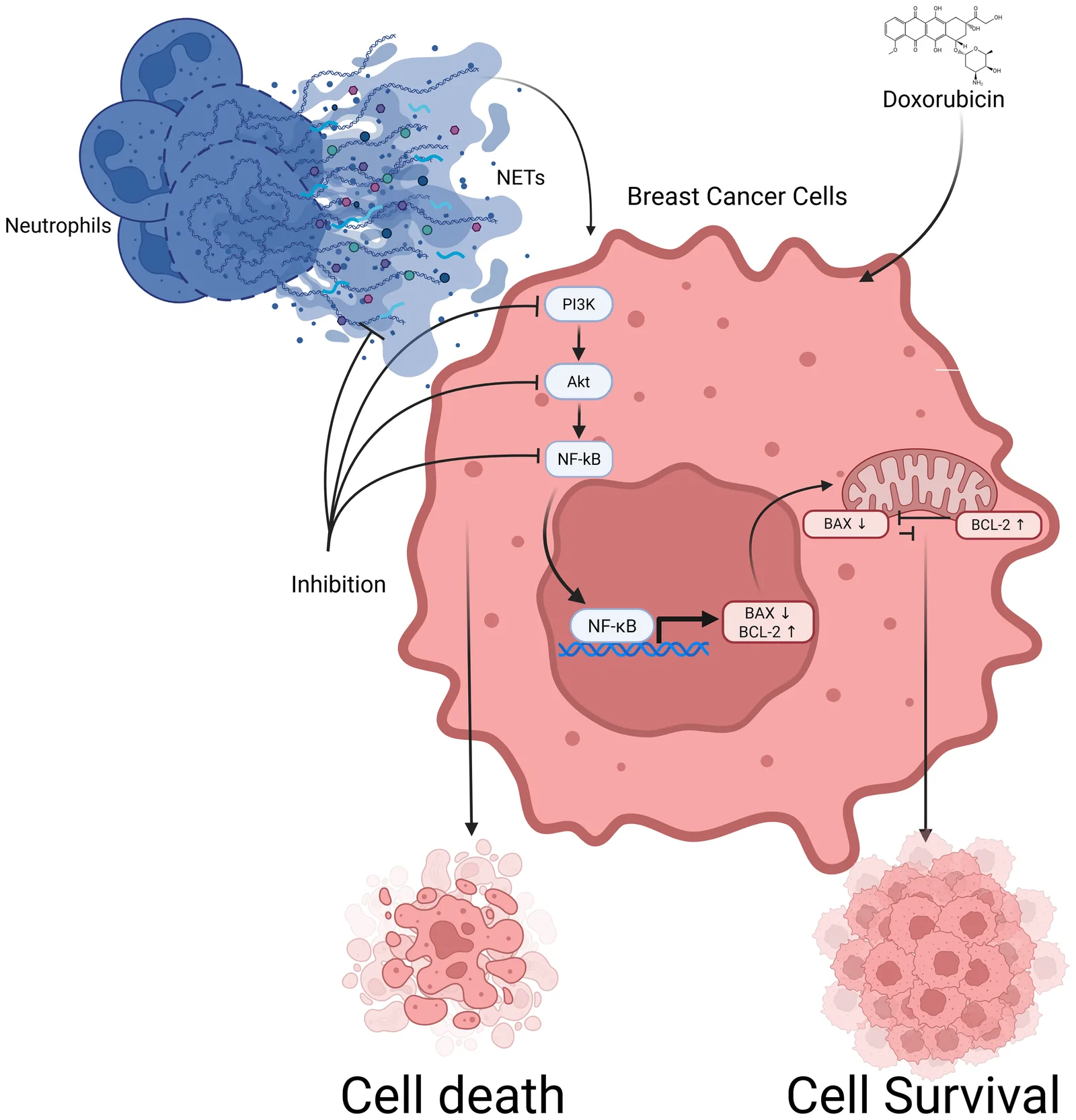
Schematic representation of NET-induced chemoresistance in breast cancer through PI3K/AKT/NF-κB activation. In our proposed model, activated neutrophils in the tumor microenvironment release NETs, structures composed of DNA decorated with neutrophil-derived proteins, that can interact with tumor cells. This, in turn, triggers the activation of the phosphoinositide 3-kinase (PI3K)/protein kinase B (AKT) signaling pathway. PI3K activation leads to AKT phosphorylation, which in turn promotes the activation and nuclear translocation of the transcription factor nuclear factor kappa B (NF-κB). NF-κB activation induces the expression of anti-apoptotic genes, such as B-cell lymphoma 2 (BCL2), while suppressing pro-apoptotic mediators, including Bcl-2-associated X protein (BAX). This disrupts the balance of the intrinsic apoptotic pathway, shifting it toward a predominantly anti-apoptotic profile and impairing mitochondrial-dependent cell death. As a consequence, NET-exposed tumor cells display reduced apoptotic signaling, increased cell viability, enhanced clonogenic capacity, and resistance to doxorubicin treatment. Disruption of NET structure by protein denaturation, pharmacological inhibition of PI3K, AKT, or NF-κB, or knockdown of AKT1 reverses this phenotype, restoring chemosensitivity. Altogether, this model identifies NET-mediated activation of the PI3K/AKT/NF-κB axis as a central driver of tumor cell survival and chemoresistance in breast cancer. Created in BioRender. de Souza, J. (2026) https://BioRender.com/664b74i.

A previous study suggested that NETs can act as physical barriers preventing chemotherapeutic agents from reaching tumor cells, particularly in dense stromal or vascularized regions (43). Beyond this passive effect, NET-associated proteases can directly activate pro-survival signaling cascades. Neutrophil elastase, for example, has been reported to activate AKT in leukemia models, promoting proliferation and suppressing apoptotic signaling (44). Furthermore, myeloperoxidase and cathepsin G promote oxidative stress and matrix remodeling that contribute to EMT and drug tolerance (29, 45). Our observations show that NETs increased Bcl-2 expression and suppressed Bax expression align with this paradigm, revealing a NET-driven shift in apoptotic balance toward survival. The relative abundance of pro-apoptotic BAX and anti-apoptotic BCL2 acts as a rheostat that sets the threshold for intrinsic (mitochondrial) apoptosis, such that a lower BAX/BCL2 ratio raises the threshold for cell death and favors survival (46). Consistent with the biological relevance of this transcriptional readout, coordinated changes in BAX and BCL2 expression have been shown to parallel directly measured apoptosis and to track with doxorubicin sensitivity in breast cancer cells, including the subtypes studied here (47). The suppression of mitochondrial apoptosis is a hallmark of chemoresistance in cancer, previously associated with Bcl-2 overexpression and NF-κB-dependent transcriptional control (48–51). By linking these intracellular mechanisms to extracellular inflammatory triggers, our results highlight how neutrophil activity can dictate drug responsiveness through integrated molecular signaling, as summarized in **Figure 9**. Of note, the magnitude of protein-level changes was generally less pronounced than that observed by qPCR, possibly reflecting post-transcriptional and translational regulatory mechanisms, as well as differences in protein stability.

The involvement of the PI3K/AKT/NF-κB pathway was further confirmed through pharmacological inhibition and AKT1 knockdown, both of which restored chemosensitivity. The PI3K/AKT signaling cascade is one of the most frequently deregulated pathways in cancer, regulating cell survival, proliferation, and therapy resistance (52, 53). Its activation triggers downstream effectors including NF-κB, a transcription factor central to inflammation and cell fate decisions (54–56), and their crosstalk contributes to tumor progression and resistance to cytotoxic therapies by preventing apoptosis and promoting DNA repair (38, 57–59). This pathway has been consistently associated with resistance to anthracyclines, taxanes, and platinum compounds across multiple tumor types (59–61). Its activation downstream of NETs exposure provides a plausible mechanistic link between inflammation and adaptive survival responses. Inflammatory mediators such as IL-8, TNF-α, and IL-1β, that can be released during NETosis, are known to stimulate these same pathways in tumor cells, leading to transcriptional reprogramming and resistance to treatments (59, 61–65). Consistent with this, Nogi et al. (66) showed that gemcitabine-treated pancreatic cancer cells secrete IL-8, inducing NETs formation through CXCR1/2 activation and establishing a feedback loop of inflammation-driven chemoresistance (66). Similar phenomena were observed in lung adenocarcinoma, where NETs-related gene signatures correlated with poor chemotherapy response and reduced overall survival (67). Whereas these studies indicate that chemotherapy can paradoxically promote NETosis, our data provide the complementary link, showing that the resulting NETs could activate pro-survival signaling and confer chemoresistance – together highlighting a feedback loop in which chemotherapy can induce NETs and limit treatment efficacy.

Chemotherapy-induced NETs formation has also been implicated in metastatic recurrence. He et al. (68) demonstrated that doxorubicin and cisplatin stimulate fibroblast senescence and NETs release in the lung, awakening dormant disseminated breast cancer cells and promoting metastatic relapse (68). These preclinical findings raise the hypothesis that therapy-induced inflammation could contribute to the late relapse observed months or years after chemotherapy, potentially through NETs-mediated remodeling of the metastatic niche – a possibility that remains to be tested in the clinic. NETs can also activate latent TGF-β and stimulate EMT-associated transcription factors such as ZEB1 (Zinc finger E-box-binding homeobox 1) and SNAIL (Snail Family Transcriptional Repressor 1), further promoting survival and invasion under cytotoxic stress (28, 29, 40). The convergence of these processes — chronic inflammation, EMT, and apoptosis evasion — creates a resilient phenotype that is highly refractory to chemotherapy.

Beyond the tumor cell-intrinsic mechanisms described here, NETs have been reported to contribute to resistance through interactions with the broader tumor microenvironment, shaping a pro-thrombotic and immunosuppressive environment together with platelets, fibroblasts, and endothelial cells (69–72). They have also been described to impair cytotoxic lymphocyte function — for instance, by sequestering CD8+ T cells and NK cells, thereby reducing immune-mediated tumor clearance during therapy and correlating with poor response to treatment across cancer types (73–75). Together, these observations pose NETs as dynamic participants in shaping therapeutic outcomes: their dual capacity to promote intracellular survival while shaping an immunosuppressive niche suggests that the tumor cell-intrinsic PI3K/AKT/NF-κB mechanism we describe here may act in parallel with immune evasion, potentially amplifying *in vivo* the chemoresistant phenotype observed *in vitro*.

Our data also provide insight into why DNase-based therapeutic strategies may not suffice to overcome NET-induced chemoresistance. Notably, DNase-mediated digestion of the DNA scaffold did not abolish the chemoprotective activity of NETs (**Figures 3A–C**), indicating that the relevant effector is retained once the DNA is removed. Thus, although DNA degradation eliminates the structural scaffold of NETs, it does not eliminate their bioactive protein cargo. NET-associated proteins such as histones, HMGB1 (High mobility group box 1 protein), and MMP-9 may remain bioactive after DNA digestion and could continue to activate TLRs or surface receptors on tumor cells (37, 42). These findings carry direct translational implications. Strategies targeting NETosis upstream — such as PAD4 inhibitors, which are currently under preclinical and early translational development for cancer therapy and have shown promising effects in limiting tumor progression and metastasis (76–79), or CXCR1/2 antagonists such as reparixin, which have demonstrated activity in breast cancer preclinical models and clinical trials in combination with chemotherapy (80–82) — may offer greater efficacy than DNA-directed approaches. Importantly, the PI3K/AKT/NF-κB axis identified here as the downstream effector of NET-driven chemoresistance is itself a clinically actionable target. Alpelisib and capivasertib, inhibitors of PI3K and AKT respectively, have received FDA approval for HR-positive/HER2-negative advanced breast cancer (83, 84), and their combination with chemotherapy in NET-rich tumor microenvironments represents a rationale worth exploring in future clinical studies. Combinatorial regimens pairing chemotherapy with such inhibitors may prevent NET-driven survival signaling and restore apoptosis, offering a mechanistically grounded strategy to improve responses in patients with chemoresistant disease (60, 85).

The present work, therefore, adds to a growing conceptual framework in which inflammation and therapy resistance are tightly intertwined. Neutrophil-derived NETs emerge as extracellular effectors capable of orchestrating intracellular survival mechanisms, linking innate immunity to oncogenic signaling. Our findings indicate that this signaling axis serves as the molecular hub through which NETs enhance drug tolerance, establishing a mechanistic bridge between inflammatory and survival cues. These results underscore the translational potential of targeting NET-related pathways to improve therapeutic outcomes.

While the present findings provide mechanistic insight into NETs-induced chemoresistance, several limitations should be acknowledged and define clear directions for future work. All experiments were conducted *in vitro*, which does not fully reproduce the complexity of the tumor microenvironment, including immune cell diversity, vascular factors, and stromal interactions. Although our results are supported by *in vivo* evidence from independent studies (32, 43, 67, 69), further validation in animal models and patient-derived material — including neutrophils from patients with breast cancer, whose NETs may differ in composition and activity from healthy-donor NETs — will be important to confirm the clinical relevance of the proposed mechanisms. Additionally, while NET-associated, heat-labile, components likely contribute to chemoresistance, the specific effector components within NETs and the corresponding tumor-cell targets responsible for initiating intracellular pro-survival signaling remain largely undefined and warrant further investigation. Proteomic analyses of NETs preparations, coupled with receptor inhibition or gene silencing, could clarify which components drive pathway activation. The pro-survival shift in BAX/BCL2 expression was not accompanied by a direct functional apoptosis assay (e.g., Annexin V/PI, cleaved caspase-3 or PARP, or sub-G1 analysis), and additional resistance mechanisms such as drug efflux were not assessed and warrant further investigation. Finally, given the heterogeneity of breast cancer subtypes and chemotherapy regimens, and the fact that genetic validation was restricted to AKT1 in MDA-MB-231 cells, future research should assess whether NETs can mediate resistance to other chemotherapeutic agents or differentially impact resistance in luminal, HER2-positive, and triple-negative contexts.

In conclusion, NETs emerge as extracellular mediators of chemoresistance in breast cancer, acting through activation of the PI3K/AKT/NF-κB signaling axis, shifting the balance of apoptosis-related factors toward survival and reducing doxorubicin sensitivity. Our findings establish a mechanistic link between neutrophil-driven inflammation and therapeutic failure, indicating that heat-labile, NET-associated components — rather than their chromatin scaffold — reprogram tumor cell survival programs across distinct breast cancer molecular subtypes. These results highlight the translational potential of targeting NETs formation, neutralizing their protein cargo, or inhibiting downstream survival signaling as complementary strategies to overcome chemoresistance and improve long-term treatment outcomes in breast cancer.

## Data Availability

The original contributions presented in the study are included in the article/**Supplementary Material**. Further inquiries can be directed to the corresponding author.

## Glossary

AKT: Protein kinase B
Bax: Bcl-2-associated X protein
Bcl-2: B-cell lymphoma 2
β-actin: Beta-actin
CD8: Cluster of Differentiation 8
CXCR1/2: C-X-C chemokine receptor 1 and 2
DMEM: Dulbecco’s Modified Eagle Medium
DMSO: Dimethyl sulfoxide
DNA: Deoxyribonucleic acid
DNase I: Deoxyribonuclease I
EMT: Epithelial–Mesenchymal Transition
FBS: Fetal Bovine Serum
GAPDH: Glyceraldehyde-3-phosphate dehydrogenase
HER2: Human Epidermal Growth Factor Receptor 2
HMGB1: High Mobility Group Box 1
HRP: Horseradish Peroxidase
IL-1β: Interleukin 1 beta
IL-8: Interleukin 8
MMP-2: Matrix Metalloproteinase 2
MMP-9: Matrix Metalloproteinase 9
MTT: 3-(4, 5-dimethylthiazol-2-yl)-2, 5-diphenyltetrazolium bromide
N1/N2: Neutrophil phenotypic subtypes
NETs: Neutrophil Extracellular Traps
NF-κB: Nuclear Factor kappa B
NK cells: Natural Killer cells
NSC: Non-silencing control (siRNA control)
PAD4: Peptidyl Arginine Deiminase 4
PAR2: Protease-Activated Receptor 2
PBS: Phosphate-Buffered Saline
PI3K: Phosphatidylinositol 3-Kinase
PMA: Phorbol 12-myristate 13-acetate
PVDF: Polyvinylidene fluoride
qPCR: Quantitative Polymerase Chain Reaction
RIPA: Radioimmunoprecipitation lysis buffer
SDS-PAGE: Sodium Dodecyl Sulfate-Polyacrylamide Gel Electrophoresis
siAKT1: Small interfering RNA targeting AKT1
siRNA: Small interfering RNA
SNAIL: Snail Family Transcriptional Repressor 1
TANs: Tumor-Associated Neutrophils
TGF-β: Transforming Growth Factor beta
TLRs: Toll-like Receptors
TME: Tumor Microenvironment
TNF-α: Tumor Necrosis Factor alpha
ZEB1: Zinc finger E-box-binding homeobox 1.
