# Supplementary material for: Neutrophil extracellular traps induce chemoresistance in human breast cancer cells through the PI3K/AKT/NF-κB pathway
Source: Front Immunol. 2026 Sep 10;17:1912140. doi: 10.3389/fimmu.2026.1912140 (PMC13600900; doi:10.3389/fimmu.2026.1912140)
Supplement: Supplementary file 1 [file Image1.pdf]

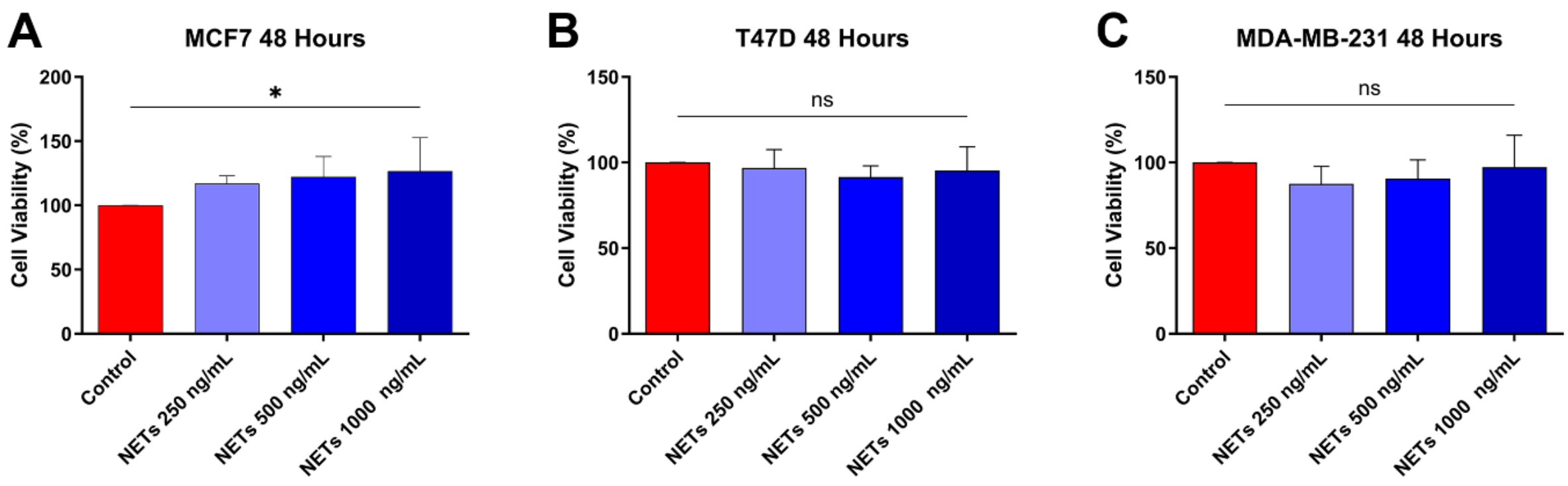

Supplementary Figure 1. NETs do not reduce cell viability after 48 h of exposure, with a pro-survival effect observed in MCF7 cells at the highest concentration tested. MTT assays were performed, with cells seeded at 10,000 cells/well in 96-well cell culture plates. Then, cells were exposed to different concentrations of NETs (250, 500, and 1000 ng/mL) for 48 h in serum-free medium. After treatment, cells were incubated with a solution of MTT. The formazan crystals generated were solubilized in DMSO, and the optical density was measured at a wavelength of 538 nm. Cell viability was expressed as a percentage of the control. Values represent mean ± SD of four independent experiments. One-way ANOVA and Tukey's post-test were used. ns, not significant; $^*P < 0.05$.
