# Supplementary material for: Neutrophil extracellular traps induce chemoresistance in human breast cancer cells through the PI3K/AKT/NF-κB pathway
Source: Front Immunol. 2026 Sep 10;17:1912140. doi: 10.3389/fimmu.2026.1912140 (PMC13600900; doi:10.3389/fimmu.2026.1912140)
Supplement: Supplementary file 2 [file Image2.pdf]

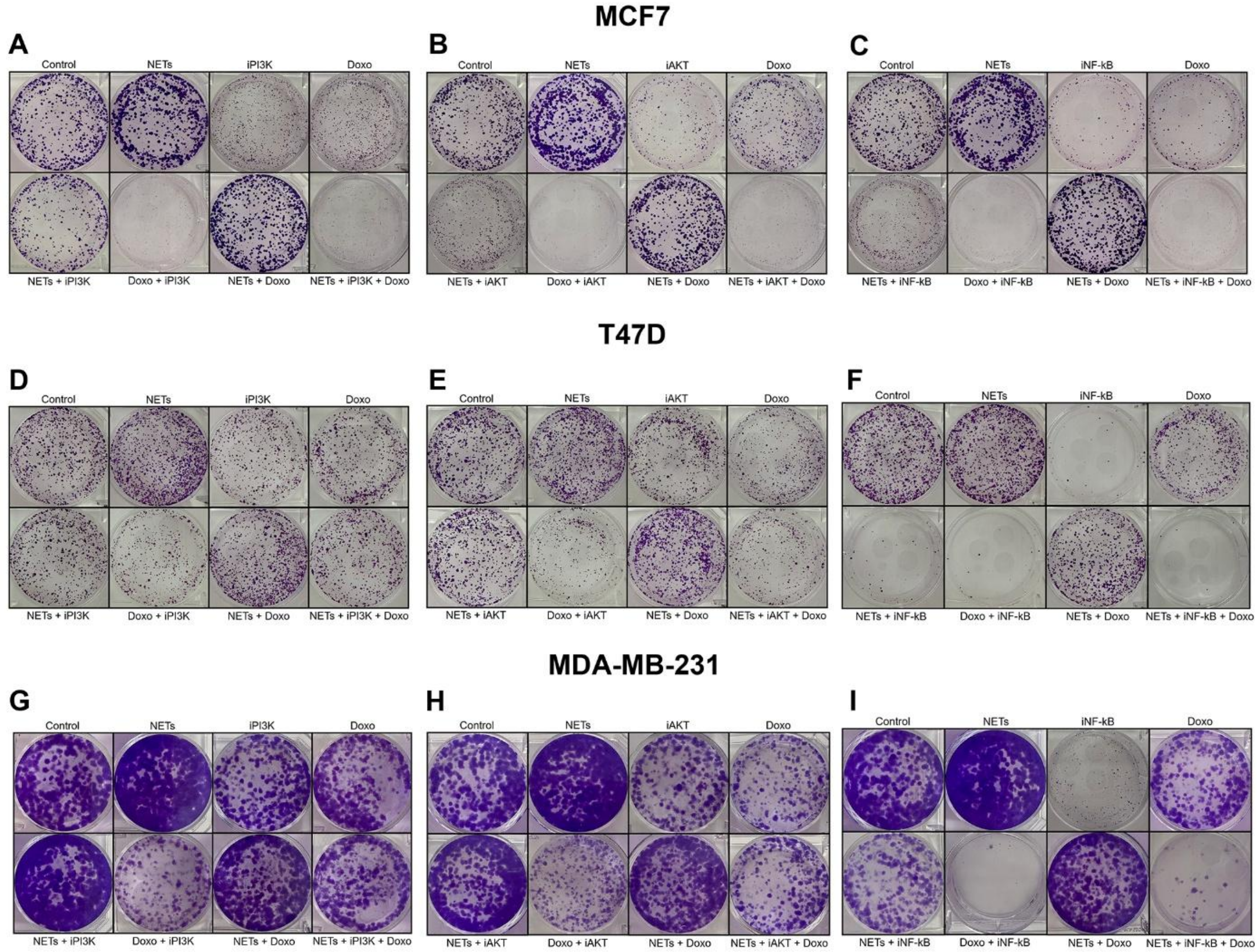

Supplementary Figure 2. Inhibition of PI3K, AKT, or NF-κB abolishes the pro clonogenic and chemoresistant phenotype induced by NETs in breast cancer cells. Representative images from three independent experiments of colonies after treatments with PI3K, AKT or NF-κB pharmacological inhibitors. Cells were seeded at a low density of 2,000 cells/well in 6-well cell culture plates. Then, cells were treated with (A, D, and G) PI3K (LY294002 – 25 μM), (B, E, and H) AKT (MK-2206 – 10 μM), or (C, F, and I) NF-κB (BAY 11-7085 – 10 μM) inhibitors 1 h prior to NETs (500 ng/mL) exposure for 24 h in serum-free medium. After stimulus, cells were treated with doxorubicin for 48 h in complete medium, then allowed to grow for 13 days. Afterwards, colonies were stained with crystal violet and photographed.
