# Supplementary material for: Neutrophil extracellular traps induce chemoresistance in human breast cancer cells through the PI3K/AKT/NF-κB pathway
Source: Front Immunol. 2026 Sep 10;17:1912140. doi: 10.3389/fimmu.2026.1912140 (PMC13600900; doi:10.3389/fimmu.2026.1912140)
Supplement: Supplementary file 3 [file Image3.pdf]

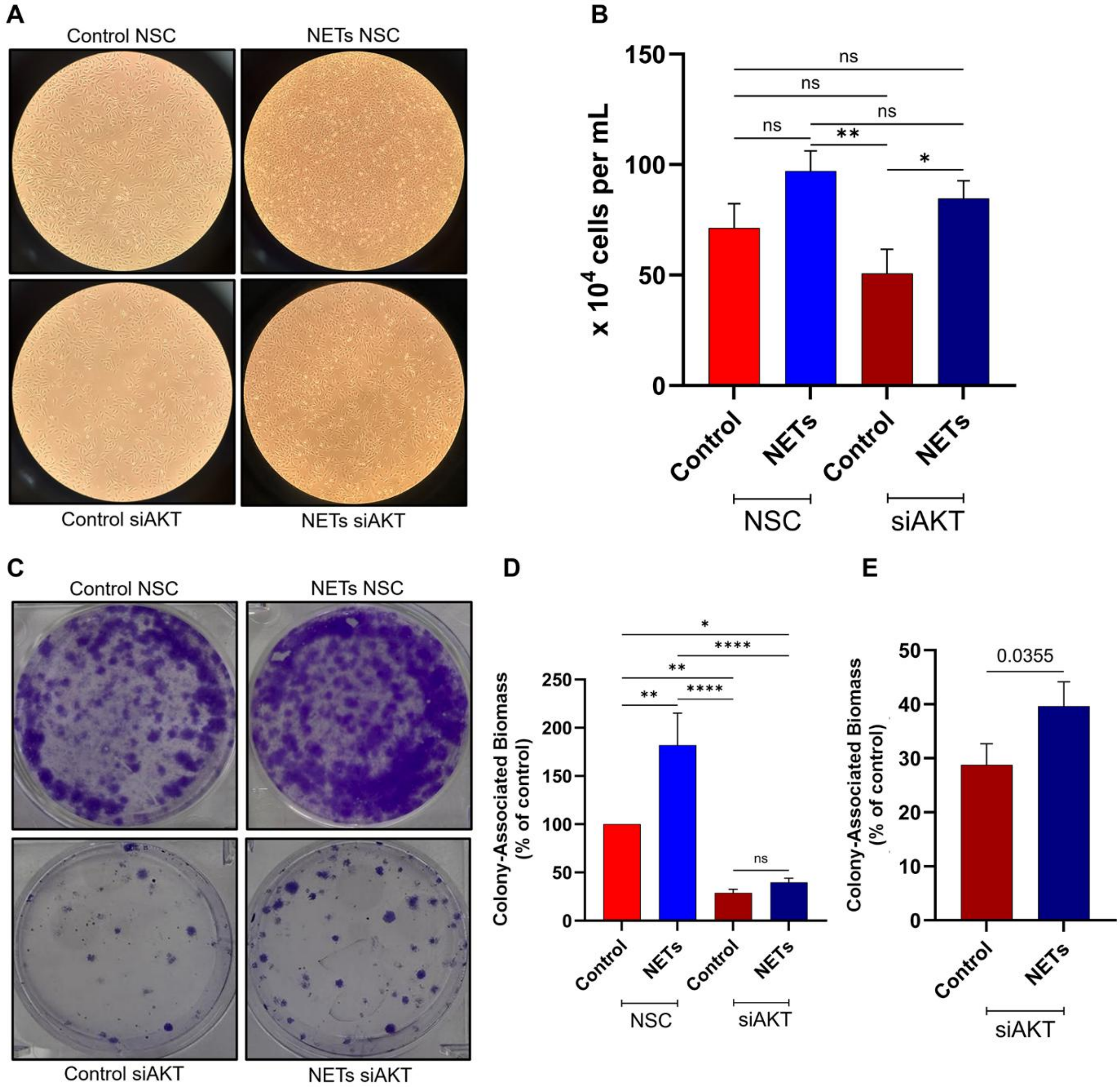

Supplementary Figure 1. AKT1 knockdown reduces NET-induced clonogenicity, yet NETs retain a residual pro-survival effect in cells with reduced AKT1 expression. To confirm the role of AKT in the observed effects of NETs, MDA-MB-231 cells were transfected with siAKT1 using Lipofectamine RNAiMAX for 48 h. At the 24-hour mark, NETs (500 ng/mL) were also added. After transfection, cells were counted and separated for each assay. NSC: non-silencing control; siAKT1: AKT1-targeting siRNA. (A) Representative image of cells after transfection and NETs treatment. Images were acquired using a 10× objective lens. (B) Quantification of the total cell number ($\times 10^4$ cells/mL in 4 mL total) after transfection and treatment with NETs. (C) Representative images from three independent experiments of colonies formed by cells transfected with NSC or siAKT1, in the presence or absence of NETs. After transfection, cells were seeded at a low density of 2,000 cells/well in 6-well cell culture plates. Then, cells were treated with doxorubicin for 48 h in complete medium and allowed to grow for 13 days. Afterwards, colonies were stained with crystal violet and photographed. (D–E) Crystal violet from colonies was eluted in methanol, and absorbance at 595 nm was measured as a readout of colony-associated biomass,

expressed as a percentage of the control. Values represent mean ± SD of three independent experiments. For cell quantification and colony formation under all conditions, one-way ANOVA and Tukey's post-test were used; for colony formation comparing two conditions, Student's t-test was performed. ns, not significant; *$P < 0.05$; **$P < 0.01$; ***$P < 0.001$; ****$P < 0.0001$.
