# Supplementary material for: Neutrophil extracellular traps induce chemoresistance in human breast cancer cells through the PI3K/AKT/NF-κB pathway
Source: Front Immunol. 2026 Sep 10;17:1912140. doi: 10.3389/fimmu.2026.1912140 (PMC13600900; doi:10.3389/fimmu.2026.1912140)
Supplement: Supplementary file 4 [file Image4.pdf]

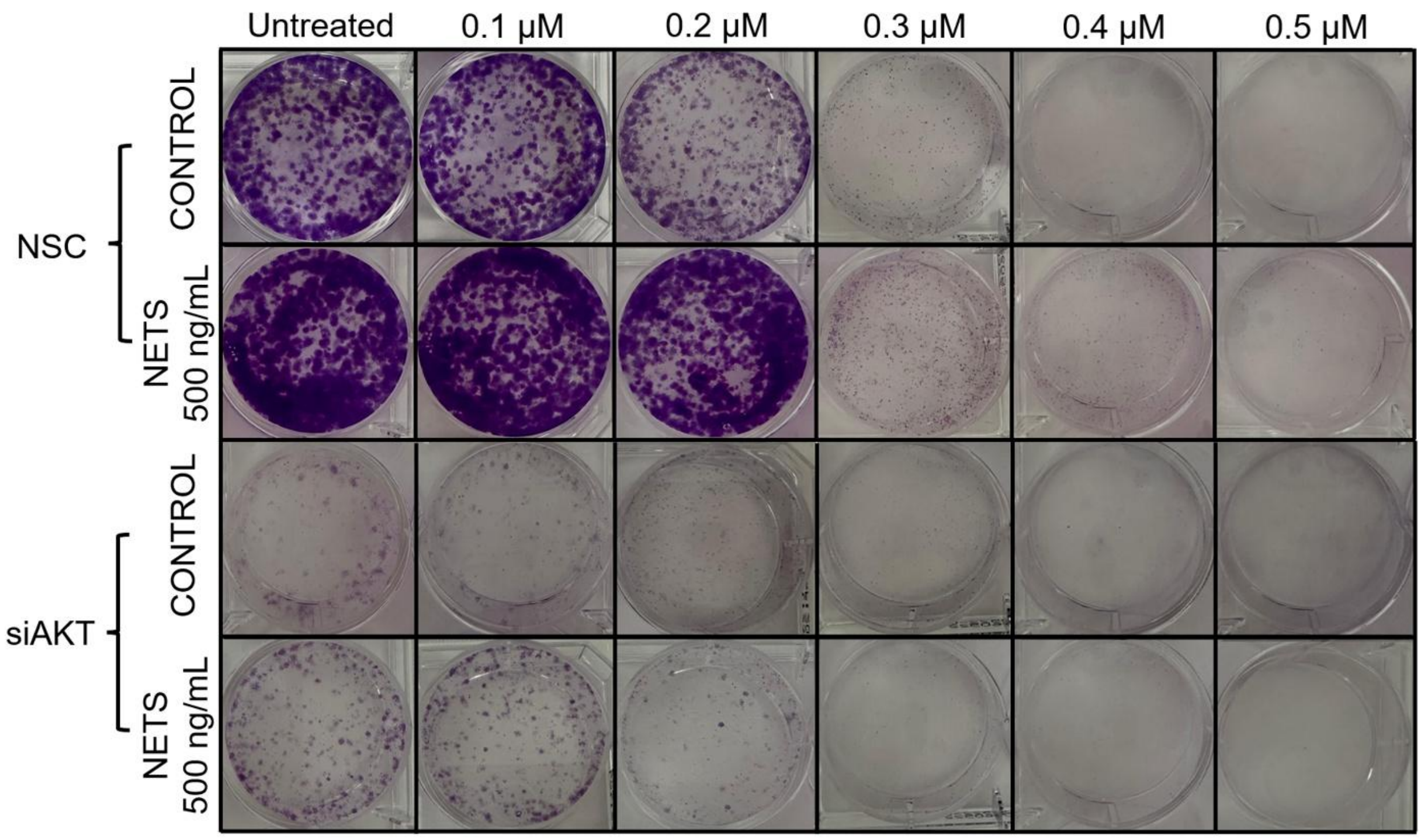

Supplementary Figure 1. AKT1 knockdown combined with doxorubicin overrides the cytoprotective influence of NETs in breast cancer cells. Representative images from three independent experiments of colonies formed by NSC- and siAKT1-transfected cells stimulated by NETs and treated with increasing concentrations of doxorubicin. To confirm the role of AKT in the observed effects of NETs, MDA-MB-231 cells were transfected with siAKT1 using Lipofectamine RNAiMAX for 48 h. At the 24-hour mark, NETs (500 ng/mL) were also added. After transfection, cells were counted and separated for the assay. NSC: non-silencing control; siAKT1: AKT1-targeting siRNA. After transfection, cells were seeded at a low density of 2,000 cells/well in 6-well cell culture plates. Then, cells were treated with doxorubicin for 48 h in complete medium and allowed to grow for 13 days. Afterwards, colonies were stained with crystal violet and photographed.
