# Supplementary material for: Neutrophil extracellular traps induce chemoresistance in human breast cancer cells through the PI3K/AKT/NF-κB pathway
Source: Front Immunol. 2026 Sep 10;17:1912140. doi: 10.3389/fimmu.2026.1912140 (PMC13600900; doi:10.3389/fimmu.2026.1912140)
Supplement: Supplementary file 5 [file Table1.docx]

**Supplementary Table 1. Primer sequences used for quantitative real-time PCR (qPCR) analysis.**

| **Gene** | **Forward (5′–3′)** | **Reverse (5′–3′)** |
| --- | --- | --- |
| *GAPDH* | TGCACCACCAACTGCTTAGG | GGCATGGACTGTGGTCATGAG |
| *BAX* | CCCGAGGTCTTTTTCCGAG | CCAGCCCATGATGGTTGTGAT |
| *BCL2* | TACAGGCTGGCTCAGGACTAT | CGCAACATTTTGTAGCATCTG |

**Supplementary Table 2. Primary and secondary antibodies used for Western blot analysis.**

| **TARGET** | **HOST-SPECIES** | **WORKING DILUTION** | **MANUFACTURER** | **CATALOG Nº** |
| --- | --- | --- | --- | --- |
| β-actin | Rabbit | 1:1000 | Cell Signaling Technology, Danvers, Massachusetts, USA | 8457S |
| β-actin | Mouse | 1:10000 | Sigma-Aldrich, St. Louis, Missouri, USA | A5441 |
| Total AKT | Rabbit | 1:1000 | Cell Signaling Technology, Danvers, Massachusetts, USA | 4691S |
| Total AKT | Rabbit | 1:1000 | Cell Signaling Technology, Danvers, Massachusetts, USA | #9272 |
| p-AKT | Rabbit | 1:250 | Cell Signaling Technology, Danvers, Massachusetts, USA | 4060S |
| Bax | Rabbit | 1:500 | Cell Signaling Technology, Danvers, Massachusetts, USA | 5023S |
| Bcl-2 | Mouse | 1:1000 | Cell Signaling Technology, Danvers, Massachusetts, USA | 15071S |
| Anti-rabbit | Mouse | 1:5000 | Dako, Glostrup, Denmark | P0448 |
| Anti-rabbit | Goat | 1:20000 | Sigma-Aldrich, St. Louis, Missouri, USA | A9169 |
| Anti-mouse | Rabbit | 1:5000 | Dako, Glostrup, Denmark | P0447 |
| Anti-mouse | Rabbit | 1:40000 | Sigma-Aldrich, St. Louis, Missouri, USA | A9044 |
